# Traumatic pulmonary pseuodocysts: two case reports

**DOI:** 10.1186/1752-1947-1-112

**Published:** 2007-10-22

**Authors:** Bulent Kocer, Gultekin Gulbahar, Nesimi Gunal, Koray Dural, Unal Sakinci

**Affiliations:** 1 Thoracic surgery, Numune Teaching and Research Hospital, Division of Thoracic Surgery, Ankara, Turkey

## Abstract

Traumatic pulmonary pseudocyst (TPP) is a rare complication, sometimes encountered after blunt thoracic trauma and even more rarely following penetrating injuries. It is more common among pediatric and young adult patients. Although TPP is usually benign in nature, complications associated with hemoptysis and secondary infection may develop. The treatment is conservative. In this report, we present two rare cases of TPP occuring after a high-speed accident and a stab wound injury, where conservative treatment provided good outcomes.

## Background

TPP as a result of a chest injury is a rare event. Santos and Mahendra presented a review in 1979 of 48 cases. Half of the 80 cases reported in the English literature have been reported in the last ten years [1-8] (Table 1). TPPs may occur after blunt trauma. There are only four TPP cases associated with penetrating trauma [9,10].

**Table 1:** The general characteristics of the TPP cases reported in the last 10 years.

Study	n	Age	Sex	Etiology	Hemo and/or pneumothorax	Treatment of TPP	Resolution time of TPP
Stathopoulos et al (2002)	1	16	Male	Motorcycle accident	1	Conservative	2 months
Melloni et al (2003)	10	27 (18–44)	Male 9 Female 1	Traffic accident 10	8	Conservative 9 Emergency lobectomy 1	5 (3–6) months
Athanassiadi et al (2003)	14	(13–24)	Male 11 Female 3	Traffic accident 14	6	Conservative	6–11 weeks
Watanabe et al (2005)	1	34	Male	Sport injury	1	Conservative	43 days
Crausman RS (2006)	1	38	Male	Industrial machinary	-	Conservative	?
Celik B and Basoglu A (2006)	1	28	Male	Mototrcycle accident	1	Conservative	?
Chon et al (2006)	12	17.7 (2–48)	Male 11 Female 1	Traffic accident 9 Fall down 2 Battery 1	11	Conservative	85.6 days
De et al (2007)	1	19	Male	Traffic accident	1	Conservative	?
Cai MH and Lee WJ (2007)	1	26	Male	Motorcycle accident	1	Conservative	?

This paper reports two cases of TPP, one of which was associated with blunt trauma and the other, with penetrating trauma.

## Case presentation

### Case 1

**Figure 1:**
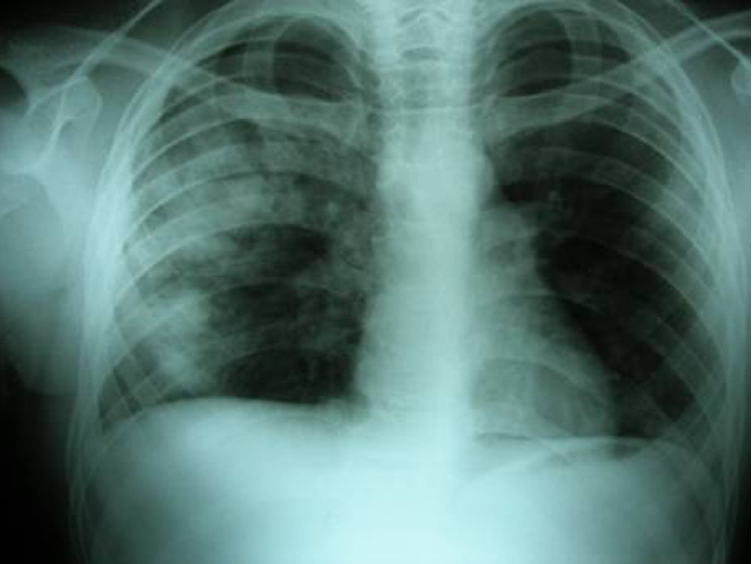
The direct x-ray image of the first patient on the 2^nd ^day of the trauma. Two cavitary lesions on the right and accompanying low-percentage pneumothorax are observed.

**Figure 2:**
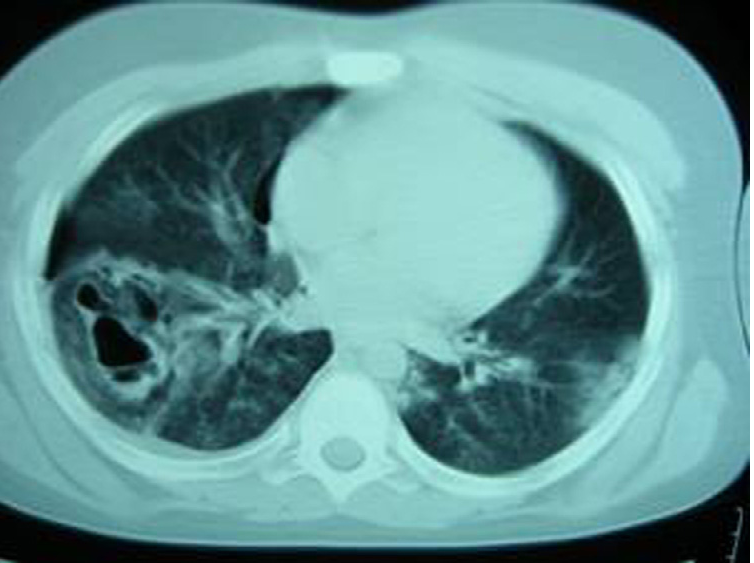
The chest computed tomography image of the first patient on the 1^st ^day, revealing multiple contusion areas and pneumothorax as well as two separate traumatic pulmonary pseudocyst.

A 13-year-old male patient presented to the emergency ward following vehicle versus pedestrian accident. The patient had complainted of right shoulder and right chest pain. On physical examination, he was hemodynamically stable and well perfused. Auscultation of the lungs revealed decreased respiratory sounds over the right hemithorax and painful right shoulder motions were noted. The white blood cell count was 19.6 K/uL, and there was a mild increase in serum transaminase, creatine phosphokinase and lactic dehydrogenase activity. The chest x-rays were consistent with bilateral parenchymal contusion and showed a fracture at the right humeral neck. The patient was admitted to our clinic. In the control x-ray taken 24 hours after admission, low percentage pneumothorax and two cavities were detected on the right (Figure 1). Thus, a chest computed tomography (CT) was obtained. The CT showed bilateral contusion, 2 cavitary lesions on the right, and minimal hemopneumothorax (Figure 2). The patient was treated by catheter aspiration and operation for his humeral neck fracture was performed on day 7. The TPP had completely resolved by the fourth week.

### Case 2

**Figure 3:**
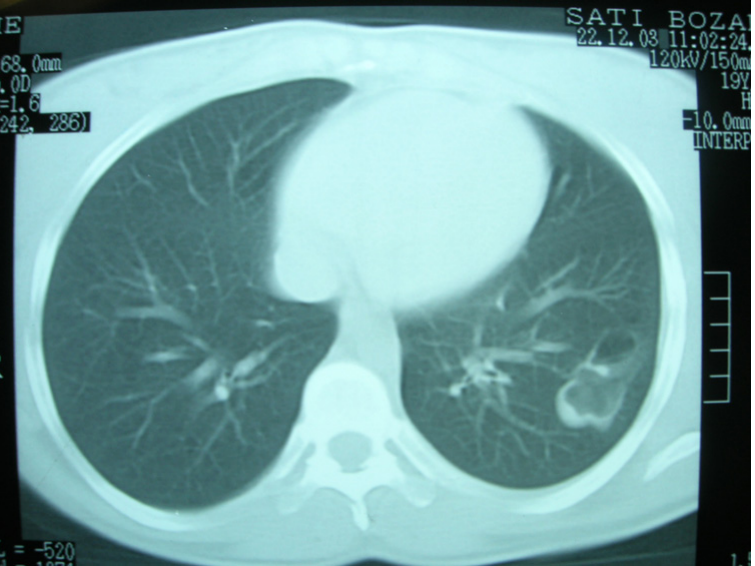
The chest computed tomography image of the second patient. A traumatic pulmonary pseudocyst.

A 24-year-old female patient presented to the emergency clinic with a stab wound injury. She was mentally alert and her heart rate was slightly elevated at 110/min. The other vital signs were normal. Respiratory sounds were diminished over her left lung. The hemoglobin count was 9.2 g/dL; white blood cell count was 11.6 K/uL and other hematologic and biochemical findings were normal. Chest x-rays of the patient revealed left hemothorax; thus, tube thoracostomy was performed on the left side. The drain was removed on the 4^th ^day and the control x-ray of the patient showed no complications. The patient was discharged on the 6^th ^day of hospitalization. On chest x-ray obtained on an outpatient basis after one month, a cavitary lesion with thick walls containing air fluid levels was observed in the left middle zone. The findings of CT evaluation were consistent with TPP (Figure 3). She was treated conservatively without any surgical intervention and empiric antibiotherapy. Subsequent x-rays at 2 week intervals showed progressive resolution of TPP. After 3 months, the x-ray showed no cystic lesion and the lesion had completely disappeared on CT imaging.

## Discussion

TPP has been defined as "pseudocyst", "cyst" or "pneumatocele". However, Santos and Mahendra proposed the term "pseudocyst" because it lacks epithelial lining [1]. In an earlier study, the incidence rates of TPPs were 0.34 % in all thoracic traumas and 2.9 % in all pulmonary parenchymal injuries [11]. TPPs can occur at almost any age, but the majority of patients are 30 years old or younger [3]. Of the cases reported in the last ten years, 85 % are male.

The significance and behavior of the TPP depend on the impact velocity, the degree of chest wall displacement, and the elasticity of the chest wall in blunt chest trauma [12]. High-velocity impact with low displacement of the chest wall (concussive forces) result in peripheral pseudocyst, while low-velocity impact with high displacement of the chest wall (compressive forces) result in central pseudocyst [12,13]. An intraparenchymal pulmonary laceration with airway disruption and leaking of air into the pulmonary parenchyma occurs in both mechanisms. The mechanism of TPP due to penetrating injury is not clearly described and requires further investigation. It may develop when air, as a result of "one way" or "check valve" mechanism, is able to enter lacerated parenchyma, but unable to escape the pleural space.

Hemoptysis, chest pain and cough were the symptoms the patients complained of and they were attributable to the pulmonary parenchymal injury but not to the TPP itself [4]. However, it may also be asymptomatic [12]. Hemoptysis may occur in up to 56% of cases [14]. Although it usually not life-threatening, in the case of massive hemoptysis, urgent thoracotomy and lobectomy may be required [3]. While our first patient had mild chest pain, our second patient was asymptomatic.

TPP can be diagnosed by chest x-ray; however, CT imaging is a more accurate method, particularly within the first days of a trauma. In a study conducted by Melloni et al within a nine-year period, none of the 10 TPP cases was diagnosed on the day of the trauma by chest x-ray, whereas the lesion in each case was demonstrated by CT [3]. Similarly, Boeuf et al detected multiple cystic structures in the contusion area of a TPP case by CT. However, no pathologies were observed in the same patient by direct x-ray imaging [15]. In the series reported by Chon et al, only one of the 12 cases was diagnosed through x-ray [2]. In the two cases presented here, TPP was not diagnosed with chest x-ray imaging on the first day. Definitive diagnosis of TPP was established and confirmed by chest CT performed after a cavitary lesion was detected in the chest x-rays.

Cavitary lesions such as cavitating hematomas, lung lacerations, and traumatic pseudocysts detected in patients presenting with trauma, may also have a non-trauma related etiology such as blebs, bullae, congenitalcysts, coccidioidomycosis, tuberculosis, hydatid disease, and pneumonia. Particularly in countries where causes of cavitation are endemic, other possible causes should be kept in mind as part of the differential diagnosis. However, clinical or radiological diagnosis of TPP is not difficult. The size, shape and nature of the wall of the TPP changes in a relatively short time, unlike other kinds of cystic or cavitary lesions. Thus, a series of chest x-rays taken over several days can be useful to differentiate TPP from other kinds of lesions, and no extensive examination is necessary [11]. The history of trauma usually delineates any confusion, but if the cavitary lesion in question does not decrease with time, other etiologies must be considered [2].

Conservative treatment of TPP is the rule, but surgery may be indicated in specific cases, such as where there is infection, bleeding or rupture into the pleural space [2]. Forty-two cases reported in the last 10 years were successfully treated conservatively except for one patient who required emergency lobectomy for massive hemoptysis [3]. Thus, although usually no specific treatment is needed, it is necessary to follow up the patient by chest x-ray until the TPP has resolved. The use of prophylactic antibiotics is unclear. Despite being the most frequent complication, secondary infection of TPP is unusual [3]. Furthermore, all of the TPP infections have been reported to occur late, and the use of prolonged prophylaxis is likely to only increase the selection of resistant organisms and promote pathogen colonization [16]. Neither of our two patients received empirical antibiotherapy treatment and no infection related findings were detected.

Average spontaneous time for radiological resolution of TPP is 3 months. An earlier study reported a mean duration of 25.3 days for spontaneous resolution in 6 non-complicated cases, while it was 145.8 days for complicated (blood filled) cases [2].

## Conclusion

In demonstration of TPP, chest CT is a more sensitive imaging method than chest x-ray. TPP may develop when air, as a result of check valve mechanism, is able to enter lacerated parenchyma, but unable to escape the pleural space. Prophylactic antibiotics are usually unnecessary. Conservative treatment is an effective way to manage TPP. However, in rare complicated cases appropriate surgical intervention may be required.

## Competing interests

The author(s) declare that they have no competing interests.

## Authors' contributions

This report reflects the opinion of the authors and does not represent the official position of any institution or sponsor. The contributions of each of the authors were as follows:

BK and GG were responsible for reviewing previous research, journal handsearching, drafting report. NG was responsible for provision of published trial bibliographies, preparing photographs. KD was responsible for quality checking, coding and classification, data processing. US was responsible for project coordination.

## Consent

The authors declare that written informed consent was received from each patient for publication of the case report.
